# Gynæcology and Obstetrics

**Published:** 1906-01-27

**Authors:** 


### GYNÆCOLOGY AND OBSTETRICS.

**Some Obstetric Complications.**—Of "eight interesting obstetrical cases" recently described by Robert Jardine,[19] five were complicated by hæmorrhage, and in three of these the bleeding was accidental. One patient was a young rachitic woman with a contracted pelvis, the true conjugate only measuring 2¼ inches. As bleeding had been free and the fœtal head was markedly overlapping the symphysis pubis, Cæsarean section was done without delay. A living child weighing seven pounds was extracted and the placenta was found partly separated. As the uterus retracted well when emptied, it was sutured with catgut and not amputated. Both tubes, however, were tied and cut. The patient made a good recovery. In another case in which severe concealed accidental hæmorrhage occurred at term, *accouchement forcé* was decided upon in preference to abdominal section and removal of the uterus, as the os was very soft and partly dilated. Manual dilatation was followed by version and extraction. Persistent uterine oozing was treated by tight plugging with iodoform gauze which was left in for forty-eight hours and may have been responsible for the diarrhœa and temperature which persisted for a fortnight. In another case of concealed accidental hæmorrhage occurring at the fifth month, the diagnosis of the doughy uterine tumour present was puzzling and lay between ordinary pregnancy with hæmorrhage and a hydatidiform mole. Dilatation and exploration of the uterus showed the nature of the condition. Unusually firm closure of the internal os and a distensible condition of the uterine wall, the result of recent pregnancy probably, combined to prevent external hæmorrhage at first. A case of placenta prævia, reported by Jardine, presents several points of interest. The patient was a primipara but had suffered from endometritis and dysmenorrhœa. The placenta-prævia was complete and delivery occurred at term. Clots were passed at intervals during the last two months of pregnancy—an unusual event in this condition—and, previous to the passage of these, a discharge of clear watery unstained fluid recurred, which was possibly amniotic fluid, for the uterus contained very little liquor later on. If it were amniotic fluid it seems that it must have escaped through the central portion of the placenta without setting up hæmorrhage. Free hæmorrhage occurred when labour commenced, and the case was treated by firm vaginal plugging for five hours, followed by dilatation with Champetier de Ribes' bag which occupied one hour, version by thrusting the hand through the placenta, and slow extraction. The child was living. It presented by the head which, on account of the small amount of liquor amnii, was firmly pressed down upon the placenta, and thus probably prevented hæmorrhage in the later months, the effused blood being retained until the ruptured sinus had thrombosed, and being then expelled in the form of clot. A case of ruptured uterus also presents some points of interest. The pelvis was generally contracted and considerable force was required to deliver with forceps. The child was alive and weighed seven pounds. An intrauterine douche was given after the placenta had been naturally delivered and returned freely. Four hours later the patient showed signs of collapse, recovered somewhat, and again collapsed with symptoms of internal hæmorrhage. The uterus was well contracted and there was no abnormal vaginal discharge. Abdominal section revealed a transverse rupture 1¼ inch long in the posterior wall of the uterus just above its junction with the cervix. The uterus was amputated. The patient ultimately recovered. Valenta[20] has tabulated the results in sixteen cases of ruptured uterus occurring in 1,350

labours—an excessively high proportion for this serious accident. Five cases recovered, three after supravaginal hysterectomy; and two, in which rupture was incomplete, after suture of the wound. No case recovered without operation. In a patient under the care of R. J. Haugh,[21] symptoms of pulmonary embolism occurred suddenly an hour after normal delivery. Dyspnœa was extreme, the right heart was considerably dilated, and in the upper lobe of the left lung numerous fine moist râles were audible. Half a pint of frothy albuminous fluid containing hæmoglobin but no red corpuscles, was coughed up. A small venesection relieved the right heart, the patient after a few hours rapidly recovered and all physical signs had disappeared sixteen hours after the onset. The rapid clearing up of signs suggested embolism due to air rather than to blood-clot, but as no intrauterine manipulations were made, the method of entry of the air is not clear, and the symptoms did not immediately follow completion of the third stage. The recorder's own suggestion is that air might have been sucked into the vagina "as the uterus was released from the somewhat powerful grasp necessary to expel a not sufficiently separated placenta." This forcible expression was employed immediately after the child was born. Thirty-four cases of that rare condition, puerperal hæmatoma without lesion of the uterus, have been collected by Whitridge Williams [22] including one of his own. By hæmatoma in this connection he means effusion of blood beneath the peritoneum into the periuterine cellular tissues. In Williams' patient, a primipara of twenty-eight, easily delivered by forceps of a small child, soon after the expulsion of the placenta there was complaint of intense tearing pain in the region of the rectum, followed in a few hours by the symptoms of acute internal hæmorrhage and hypogastric swelling. The uterus was well contracted and there was no vaginal loss. At the operation the uterus and its appendages were seen to be quite normal, but much blood-clot was found infiltrating the cellular tissue of the pelvis and extending to the layers of the abdominal parieties. The bleeding was traced to an oozing surface on the antero-inferior aspect of the bladder. The supravaginal portion of the cervix was not lacerated. The patient recovered. Williams thinks in the majority of these cases expectant treatment may be adopted, but if there are signs of continued hæmorrhage the abdominal is safer than the vaginal route for operation. A case of precipitate labour in which the mother, a primipara, was found dead, apparently from the shock of a ruptured uterus, sudden diminution of intra-abdominal pressure, and probably actual hæmorrhage, and the fœtus was also dead owing to the circumstances of its birth, has been recorded by Rayner and Stuart.[23] The publication of the gruesome details of the case suggests questions as to precipitate and unconscious labour of much medicolegal interest. The phenomenon is probably caused by a combination of factors. Winckel [24] regards as the most constant excessively powerful pains, multiparity, small or macerated fœtus, short cord, position of the mother at the time of delivery, and the presence of epilepsy, bronchitis, or other general affection in her. Prolapse or inversion of the uterus is more common than rupture. Two cases of pregnancy complicated by uterine cancer [25] have been recorded. In one with extensive inoperable growth a Cæsarean-Porro operation was done at the onset of labour and a living fœtus, which, however, died in three days, was removed. The patient died on the eighteenth day of uræmia. In the other, in which growth was limited to the cervix, Cæsarean section was followed by total hysterectomy. The child throve and the mother remained well for eighteen months. Death from recurrence occurred three years after the operation.

[19] Glasgow Med. Jour., August. [20] Edinburgh Med. Jour. July, p. 82. [21] Lancet, July 29, p. 289. [22] Brit. Med. Jour. June 17, p. 1345. [23] Lancet, June 17. [24] Ibid., July 1, p. 37. [25] Ibid., Oct. 7, p. 1037.

